# Should UK Pneumococcal Vaccine Eligibility Criteria Include Alcohol Dependency in Areas with High Alcohol-Related Mortality?

**DOI:** 10.3390/vaccines6020025

**Published:** 2018-05-02

**Authors:** John D. Mooney, Michael Imarhiagbe, Jonathan Ling

**Affiliations:** 1 School of Nursing and Health Sciences, University of Sunderland, Sunderland SR1 3SD, UK; bg51vv@student.sunderland.ac.uk (M.I.); jonathan.ling@sunderland.ac.uk (J.L.); 2 Directorate of Public Health, Sunderland City Council, Sunderland SR2 7ND, UK

**Keywords:** pneumococcal vaccines, risk factors, alcoholism, alcohol use disorder, mortality

## Abstract

A recently reported steep increase in the incidence of invasive pneumococcal disease (IPD) in adults in the North East of England was primarily associated with pneumococcal sero-types found in the 23-valent pneumococcal polysaccharide vaccine (PPSV23). This region also has one of the highest rates of alcohol-related premature mortality and morbidity in the UK. Given that alcohol dependence is long acknowledged as one of the strongest risk factors for IPD mortality, we feel there is an increasingly compelling case to look again at the divergence of UK vaccine guidance from that of the World Health Organisation and the Centre for Disease Control in the USA, in the non-inclusion of alcoholism as an indicator condition that would potentially benefit from receiving PPSV23 vaccine. Such a re-think would represent a responsible evaluation of vaccination guidance in the face of newly emerging epidemiological findings and would have the potential to save lives in a very marginalised and vulnerable section of the population. We propose therefore that alcohol dependency (now referred to as alcohol use disorder), should be re-considered an indicator condition for receiving pneumococcal vaccine in North East England, where mortality from pneumococcal disease has been rising and which already has an excessive burden of alcohol-related mortality.

## 1. Background Context

Alcohol use disorder (AUD) is the most important risk factor in adults of working age (<65) for invasive pneumococcal disease (IPD), a serious and often life-threatening infection [1]. Previously referred to diagnostically as alcohol dependence, AUD (or alcoholism) significantly increases mortality for those affected by the disease [2]. With recent increases in adult pneumococcal disease incidence in North East England [3] combined with long established elevated rates of alcohol-related mortality in this region [4], we argue that there is a persuasive case for looking again at the current recommended prevention measures, including the potential inclusion of AUD as indicator criterion to receive the 23-valent polysaccharide pneumococcal vaccine (or PPSV23, as described below). Given the high regional burden of alcohol-related disease alongside the increased incidence of IPD in North East England, a properly evaluated regional modification in vaccine eligibility to include AUD, even for a trial period, should be highly informative around the likely benefits or otherwise, especially as we are now seeing a rapid rise in non-PCV13 vaccine serotypes in England as a whole [5].

## 2. Current Vaccine Guidance in the UK and Internationally

In order to reduce the significant public health burden associated with the more severe clinical manifestations of pneumococcal infection, there are two types of vaccine in current use [6]:

Pneumococcal conjugate vaccine (PCV13 or Prevnar 13^®^): recommended for infants from two months of age as part of the routine childhood immunisation schedule and pneumococcal polysaccharide vaccine (PPSV23 or Pneumovax23^®^), recommended as a single dose for adults over 65 years and ‘at-risk groups’ aged two years or over.

For those at risk of IPD because of underlying alcohol-related disease, the UK Department of Health’s ‘Green Book’ advises that the PPSV23 vaccine is relatively ineffective in ‘alcoholism’, although it is recommended for individuals with chronic liver disease as an appropriate ‘clinical risk group’ [6]. In not specifically recommending PPSV23 vaccine for those with alcoholism, the current UK guidance is at variance with that from the USA and the World Health Organisation [7], as well as a number of other European countries with a high public health burden due to alcohol, such as Finland and Ireland [8].

## 3. Epidemiology of IPD and Vaccine Implications

While the effectiveness of the newer conjugate PCV13 vaccine has recently been confirmed in protecting older adults from ‘vaccine type’ IPD [9], it’s more restricted range of sero-groups versus the greater sero-group diversity seen in adults in general has restricted the scope for its widespread adoption in adults. It is also the case that incidence in older children and adults (from ages 5 to 64), in North East England has now returned to levels seen before this introduction (although the incidence in infants remains reduced) [3]. Indeed, a recently published analysis for England and Wales concluded that the rapidly increasing incidence in non-PCV13 vaccine serotypes in all regions was at risk of “compromising the success” of the current vaccination programme [5]. Other factors strengthening the case for a re-appraisal of PPSV23 guidance are firstly that recent rises in IPD have been largely attributable to PPSV23-exclusive serotypes [3], together with epidemiological data linking those who misuse alcohol with a higher risk of contracting one of the more lethal (non- PCV13) serotypes such as 11A and 19F [10,11]. While recent studies of PPSV23 effectiveness, including a sub-analysis of high risk individuals in high income countries [12], have not been especially compelling, there were doubts expressed by the investigators over whether their analysis had been sufficiently powered for these results to be conclusive. 

With specific regard to risk factor epidemiology, an international review has confirmed that the risks of IPD for individuals with two or more co-morbidities are equivalent to those in the highest risk group such as the immunocompromised [13]. In addition, a recent large-scale revaluation of risk categories in the USA found that adults with ≥2 concurrent comorbid conditions had pneumococcal disease incidence rates that were as high as, or higher than, rates observed in those with traditional high-risk conditions [14]. Since high rates of chronic disease co-morbidities cluster in more socially deprived communities, of which there is a high and persistent concentration in North East England [15], the case for implementing additional protection against a vaccine-preventable, life-threatening disease in a high risk group (known to be over-represented in this region), is we believe, increasingly persuasive.

## 4. Practicalities and Effectiveness of PPSV23 for Those with Alcohol Use Disorder

Since individuals who misuse alcohol are a notoriously difficult sub-group of the population to engage within mainstream health services [16], a recent feasibility project within North East England evaluated the prospects for distributing and administering vaccines within community and health and social care settings, including high street pharmacies, residential hostels, or hospital A & E departments [17]. While no setting was without logistical challenges, there was nevertheless a willingness to explore how best to overcome access barriers to receiving vaccination. Of course, identifying those who would be most likely to benefit from a revision to the guidance has its own challenges: while there are clear clinical criteria for diagnosing AUD in the relevant UK NICE (National Institute for Clinical Excellence) Guidance [18], the same document also highlights the issue that alcohol problems are very under-recognised and under-reported in UK primary care [19]. Perhaps the most easily identifiable group to offer PPSV23, due to their elevated risk status because of drinking, would be those referred to specialist alcohol services [18] and those identified as being at risk of moderate to severe AUD from receiving a high score on AUDIT, the WHO-developed screening test, or the ‘alcohol use disorder identification test’ [20].

In terms of likely cost effectiveness, all 11 included studies in a recent review [21] found that vaccination with PPSV23 was cost-effective, and in some cases a cost-saving strategy for the prevention of IPD, an effectiveness which rose with increased case fatality (as is the case for those with alcoholism [2]). While another direct economic comparison of PPSV23 with PCV13 conjugate vaccine in the USA found the conjugate-based strategy to be dominant [22], this overlooked the scenario (as is currently the case in North East England [3], and increasingly for the rest of the UK [5]), that predominant strains were not included in PCV13 but were included in PPSV23. In the latter cost effectiveness analysis, the very conservative cost per QALY (i.e., quality-adjusted life year) (given the assumptions about predominant serotypes), for adults with co-morbid conditions, was $34K (or £25.5K). This would be well within the UK’s National Institute for Clinical Excellence (NICE’s) widely acknowledged range for acceptable cost effectiveness of between £20K to £30K per QALY [23].

Cruickshank and colleagues have also drawn attention to the divergence in PPSV23 vaccine guidance relating to alcoholism between the UK and other national jurisdictions, as well as highlighting the potential for its likely effectiveness in this group [24]. While they were ultimately unconvinced of the added benefits that a change in vaccine policy might confer (given that those with established chronic liver disease are already included), they do stress the importance of ongoing vigilance around the prevailing epidemiological evidence in deciding on such a policy change. As highlighted above for this region, IPD incidence caused by non-PCV13 serotypes has been rapidly increasing, a phenomenon now also observed across the whole of England and Wales [5]. 

## 5. Conclusions and Next Steps

In conclusion, we propose that the recently increased IPD incidence in North East England that is largely attributable to pneumococcal sero-groups contained within PPSV23 (and not in PCV13) makes a persuasive case to increase efforts to ensure that all high-risk groups are targeted with this effective vaccine. Given the additional links between alcohol misuse and the more lethal IPD serotypes [10,11], we consider that North East England, already acknowledged to have one of the highest burdens of alcohol-related premature death [4] (in combination with high levels of other relevant co-morbidities [15]), might represent a worthwhile testing ground for a reappraisal of the vaccine guidance, namely to include those with alcoholism within the target group recommended to receive PPSV23 vaccine.

Although the inconclusive nature of some of the evidence around PPSV23’s protective efficacy in those with alcoholism or AUD means that there are probably insufficient grounds at present for a UK-wide revision of vaccine guidance, some form of experimental trial with matching English region(s) of equivalent alcohol impact as controls (such as the North West), would constitute an informative and highly policy relevant intervention using a quasi-experimental design, the benefits of which have recently been summarised by Wilder-Smith et al. [25]. Such a trial intervention would need to be accompanied of course with enhanced surveillance protocols for diagnosis, serotyping, and confirmation of vaccination status in the participating regions. Naturally, the findings derived from any such trial would have the potential to inform modifications to policy in many countries, whether they were found to be supportive or not of including alcoholism as an indicator condition for to receive the PPSV23 vaccine. In either case, the rapidly evolving epidemiology and changing risk profile of this serious pathogen, reminds us very much of the need to be cognisant of variations in infectious disease distribution as well as the need for vaccination policies to be more regionally responsive, where there is a credible case for public health benefit.
